# Effect of ionic tooth brushes on gingivitis among Indian patients undergoing orthodontic treatment

**DOI:** 10.6026/9732063002001645

**Published:** 2024-11-30

**Authors:** Simran Saluja, Rupali Kalsi, Kumar Saurav, Gunjan Gupta, Sachit Anand Arora, Vandana K.L, Shivesh Kumar Mishra

**Affiliations:** 1 Department of Periodontics, ITS Dental College, Hospital and Research Centre, Greater Noida, Uttar Pradesh, India; 2 Department of Dentistry, Government Institute of Medical Sciences, Greater Noida India; 3 Department of Periodontics, Santosh Dental College, Ghaziabad, Uttar Pradesh, India; 4 Department of Periodontics, College of Dental Sciences, Davanagere, Karnataka, India

**Keywords:** Ionic toothbrush, orthodontic toothbrush, gingivitis, fixed orthodontic treatment, plaque control

## Abstract

The aim of the study was to evaluate and compare the efficacy of ionic toothbrushes versus orthodontic toothbrushes on gingivitis in patients undergoing orthodontic treatment. The study included 50 patients who were divided into 2 groups: Ionic and Ortho toothbrush users group. The clinical parameters like gingival bleeding index, plaque control record, gingival enlargement index and patient hygiene performance were recorded at baseline, 7 days, 21 days, 45 days and 90 days. It can be concluded that ionic toothbrushes give improved results than orthodontic toothbrushes in terms of maintaining oral health in gingivitis patients undergoing fixed orthodontic treatment.

## Background:

Maintaining optimal oral hygiene is a prerequisite for healthy dentition and overall well-being. With adequate measures and compliance, certain factors like tooth malalignments, carious lesions and oral appliances make the removal of plaque altogether more challenging. Gingivitis is a revocable condition which is associated with bacterial plaque. It usually settles down in 1 week after the re-initiation of oral hygiene procedures. If left neglected, gingivitis will gradually advance to periodontitis [1]. Thus, oral hygiene maintenance is more crucial for patients undergoing orthodontic treatment; any breach in maintenance can have undesirable Sequelae like gingivitis, periodontitis, dental caries etc. The primary materials used in this therapy include brackets, tubes, band materials, ligating materials and archwires. However, these materials tend to attract microbial adhesion, significantly impair oral hygiene and create new areas for plaque and debris retention. Consequently, this increases the risk of microbial build-up and potential infection for the wearer [2]. The appliances also protect the plaque from the effects of brushing, mastication and salivary flow [3]. It is evident that orthodontic forces are not detrimental to periodontal tissues in patients with good oral hygiene, whereas, in the presence of plaque, they may lead to angular bone loss, which can contribute to undesirable sequelae of orthodontic treatment [4, 5-6]. Thus, adequate oral hygiene is primely important in patients undergoing orthodontic treatment. Research has suggested that orthodontic toothbrushes are superior in comparison to conventional toothbrushes in maintaining the oral hygiene of patients with fixed orthodontic appliances [7]. Conventional, Powered and orthodontic toothbrushes work on the principle of mechanical cleansing on tooth surfaces. On the other hand, Ionic toothbrushes have dual action; apart from physically removing plaque and debris from the teeth, they use an electric charge to disrupt and hinder plaque formation [8]. We hypothesize that ionic toothbrushes may be more beneficial in maintaining the oral hygiene of subjects undergoing fixed orthodontic treatment than orthodontic toothbrushes. There is an abundance of studies comparing powered and manual toothbrushes, but there is a paucity of literature comparing ionic toothbrushes to a manual toothbrush. Hence, the aim of the study was to evaluate and compare the effect of ionic toothbrushes on gingivitis and plaque in patients undergoing orthodontic treatment.

## Methods and Materials:

## Study design:

A double-blind, randomized clinical trial was structured wherein the examiner and the microbiologists were unaware of the distribution between the two groups. After getting approval from the institutional ethical committee (reference number IEC/Perio/3/21), a group of 56 individuals aged between 14 -25 underwent screening and were recruited, among which 6 were lost during follow-up. Eventually, the study was carried out for 90 days in 50 subjects, wherein 26 subjects were given Ionic toothbrushes (*Ionic Kiss* TM) and 24 subjects were given ortho toothbrushes (*Stim Ortho* MB). Similar oral hygiene instructions and motivation were given by a single examiner to all the participants. The inclusion criteria encompassed patients of both genders, subjects undergoing orthodontic therapy and patients with gingivitis as per the AAP 2017 classification. The exclusion criteria comprised of participants having systemic conditions, with familial predilection of periodontal disease, retained deciduous teeth, less than 20 teeth, patients who had received periodontal therapy 6 months before the study, smokers or former smokers, pregnant and lactating women, participants with periodontal pockets or CAL. The study was carried out in full accordance with the ethical principles of the World Medical Association Declaration of Helsinki. An informed consent was taken from all the patients before their participation in the study.

## Sample size derivation:

The formula for calculating sample size is-

n = 2 s2(Z1+Z2)2/((M1-M2))

The minimum sample size calculated was 50 and we proposed to take a sample size of 56, which would yield 80% power to detect significant differences, with an effect size of 0.82 and a significance level of 0.05

## Clinical measurements:

The study was designed for 90 days and indices were recorded at 0, 7, 21, 45 and 90 days. Scoring of Gingival Bleeding index (Ainamo & Bay, 1975) [9]. Gingival Enlargement Index (Bokenkamp and Bohnhorst, 1994) [10], Plaque Control Record (O'Leary, Drake and Naylor, 1972) [11] using a two-tone disclosing agent (Alpha Plac) and Patient hygiene performance (A.G. and Haley J.V, 1968) [12] was done. During each recall visit, all the clinical parameters were scored.

## Collection of samples:

Plaque sample collection for microbiological assessment to detect Colony Forming Units (CFU) comprised of *Streptococcus mutans*, *Actinomyces viscosus* and *Veillonella parvula* was done on 0 and 21st day. The area was isolated with cotton pellets and Gracey curettes were placed into the gingival sulcus to obtain plaque samples. The plaque samples were transferred into an Eppendorf tube with a reduced transport fluid medium. All samples were labeled and sent to the laboratory within 72 hours after sample collection.

## Statistical analysis:

Statistical Package for the Social Sciences (SPSS) was used to analyze the data. Statistical analysis was done using descriptive statistics tools such as Mean and SD for representing quantitative data. Probability p < 0.05 considered as significant. An unpaired t-test was used to find significant differences between both groups for parametric distribution data. Paired t-test was used to find significant differences within each group for parametric distribution data. Mann Whitney U test was used to find significant differences between both groups for non-parametric distribution data. Kruskal Wallis H test was used to find significant differences within each group for parametric distribution data. The Chi-square test was used to find statistically significant intergroup and intragroup comparison differences for percentage/proportion data.

## Results:

Table 1 shows the comparison of clinical parameters between the ionic and manual toothbrush groups. Significant differences were observed between the groups on the 45th and 90th days for the mean gingival bleeding index. There was a statistically significant change in the gingival bleeding index from baseline to the 90th day on intragroup comparison within the Ionic toothbrush group. Regarding the mean gingival enlargement index, significant differences were noted between the groups on the 21st, 45th and 90th days. There was also a significant change within the ionic toothbrush group from baseline to the 90th day. For the mean patient hygiene performance index, significant differences were found between the groups on the 21st, 45th and 90th days, with a notable intragroup difference in the ionic toothbrush group from baseline to the 90th day. Similarly, for the plaque control record, significant differences were observed between the groups on the 21st, 45th and 90th days and the Ionic toothbrush group showed a significant change from baseline to the 90th day.

As Table 2 depicts, there was a statistically insignificant decline in mean streptococcus mutans count from baseline to the 21st day for both groups. Whereas the intergroup comparison was made, there was a statistically significant reduction in the group's S.mutans count on the 21st day using ionic toothbrushes. The mean decrease in *Actinomyces viscosus* count from baseline to the 21st day was statistically significant for the ionic toothbrush users. On the contrary, its mean count reduced but was not statistically significant for the ortho toothbrush group. The intergroup comparison was also statistically significant on the 21st day. The reduction in mean Veillonellaparvula count from baseline to the 21st day for the ionic group was statistically significant, while there was a statistically insignificant reduction for the ortho toothbrush. The intergroup comparison revealed a statistically significant decrease on the 21st day using ionic toothbrushes.

## Discussion:

Formation of dental plaque is inevitable in the oral cavity and its control becomes a herculean task in patients wearing fixed orthodontic appliances. The aftermath of poor plaque control in this category of patients is more alarming. The first pointer of oral hygiene assessment is dental plaque, which was assessed qualitatively and quantitatively in our study. Plaque control record is a method of recording the presence of the plaque on individual tooth surfaces. In this study, the focus was on early plaque colonizers like *Streptococcus mutans*, *Actinomyces* and *Veillonella species*. Streptococci are considered early colonizers because of the proteins expressed on their surfaces; these adhesins enable interactions with salivary, serum and extracellular matrix components, host cells and other microbes. *Streptococcus mutans* play a key role in causing dental caries due to their ability to adhere to the enamel salivary pellicle and other plaque bacteria. Streptococci and lactobacilli are potent acid producers, leading to an acidic environment that increases the risk of caries [13, 14-15]. Conflicting literature is available stating the mode of action of ionic toothbrushes. The reasonable explanation for this phenomenon could be that an ionic toothbrush interferes with the initiation of plaque formation and thus, its subsequent maturation. It is noteworthy that mechanical tooth brushing decreases the thickness of the pellicle, but it does not completely remove it from the tooth surface [16]. Normally, pellicle formation marks the first stage of plaque formation, after which bacteria's initial colonization occurs [17]. The tooth surface is negatively charged and by this, it selectively adsorbs positively charged salivary glycoproteins, which are followed by colonization of negatively charged initial colonizers, embarking biofilm formation. While brushing with ionic toothbrushes, the tooth surface is cleaned mechanically, decreasing the pellicle thickness and along with this, it is influxed with negatively charged ions, which subsequently changes polarity, making it non-conducive for the attachment of early colonizers. Previously, it is documented that in the initial stages of plaque formation, bonding between plaque Pellicle and initial colonizers is mediated by calcium ions wherein there is Ca2^+^ bridge formation takes place between these two [8]. But the negatively charged ions anions influx by ionic toothbrushes inhibits the formation of this bond. Therefore, this helps minimize the plaque mass on the surfaces of the teeth, which is observed in our study, too. Similar results were reported by Otani *et al.* [18] and Deshmukh *et al.* [8] using Ionic toothbrushes in their respective studies. On the contrary, a study was done by Maki *et al.* [19]. Where they reported that there were no changes in the oral microorganism levels at the end of the study, no matter what type of toothbrush was used. There was a statistically significant decrease in bleeding scores in ionic toothbrush users for the mean gingival bleeding index. Gingival bleeding is an objective sign of inflammation in the gingival connective tissues. Bleeding on probing is an effective method of checking for gingivitis. It is superior to color changes when diagnosing a case of gingivitis. The microbiota responsible for the gingivitis is Red Complex, *i.e.*, *Porphyromonasgingivalis*, *Treponemadenticola* and *Tanarella forsythia*, which colonizes during plaque maturation after initial colonization. The Ionic action of toothbrush and its inhibitory action on plaque formation and its maturation seems to decrease the red complex of microbiota and thus bleeding on probing scores. Thus, this appears to be the reasonable explanation for decreased bleeding scores and gingival enlargements for ionic toothbrush users in our study. Similar results are reported by Deshmukh *et al.* [8]. However, Singh *et al.* [20] reported that the sonic toothbrush was insignificantly superior to the ionic toothbrush. According to a study by Maki *et al.* [19] ionic toothbrush was significantly more effective in plant hygiene. They concluded that the lithium battery ionic toothbrush removed plaque more efficaciously. There was a decrease in all the clinical and microbiological markers of gingival disease in both groups, but the results were significantly better in ionic toothbrush users. In both groups, oral hygiene instructions were communicated by the investigator on day one and orthodontic and ionic toothbrushes were distributed to the respective subjects. Thus, oral hygiene instructions and the unique design of orthodontic toothbrushes with smaller brush heads and bristle arrangements may contribute to improved plaque scores and gingival health in the comparator group.

## Conclusion:

Optimal oral hygiene is mandatory for patients undergoing orthodontic treatment. It is more important per se for patients undergoing orthodontic treatment as orthodontic appliances compromise the maintenance and make tooth surfaces plaque retentive. Based on the inferences of the present study, it can be concluded that ionic toothbrushes produce better results in terms of maintaining good oral hygiene than orthodontic toothbrushes. The use of ionic toothbrushes enhances plaque control by its ionic action. With previous studies, orthodontic toothbrushes have proved to be superior than manual toothbrushes owing to smaller brush heads and shorter bristles in the center, allowing them to better adapt to the specific surfaces of teeth with fixed orthodontic appliances. However, our study found that ionic toothbrushes are more efficient in maintaining oral hygiene. In the future, a novel ionic toothbrush with a brush head in the design of an orthodontic toothbrush may prove to be a valuable addition to the oral health devices designed for this category of subjects.

## Figures and Tables

**Table 1:** Comparison of the clinical parameters in the ionic group and ortho group

**Clinical Parameters**	**Time Interval**	**Ionic Group N= 25**	**Ortho Group N=25**	**Intergroup Comparison**
		**Mean ± SD**	**Mean ± SD**	P value
Gingival Bleeding Index	At Baseline	52.88 ± 6.34	51.96 ± 12.13	0.737
	7th day	45.93 ± 7.15	47.68 ± 7.25	0.396
	21st day	37.54 ± 7.56	41.7 ± 8.85	0.08
	45th day	27.68 ±7.0	38.3 ± 9.42	<0.001 **
	90th day	19.4 ± 4.6	34.78 ± 12.38	<0.001 **
Intragroup comparison	Group I (Ionic): Paired t-test value = 27.5, p< 0.001**			
(Baseline vs 90th Day)	Group II (Ortho): Paired t-test value = 14.6, p=0.006*			
Gingival Enlargement Index	At baseline	1.08 ± 0.4	1.2 ± 0.4	0.299
	7th day	1.0 ± 0.28	1.16 ± 0.37	0.097
	21st day	0.4 ± 0.5	0.96 ± 0.35	<0.001 **
	45th day	0.0± 0.0	0.32 ± 0.47	0.002*
	90th day	0.0± 0.0	0.28 ± 0.45	0.004*
Intragroup comparison	Group I (Ionic): Paired t-test value = 25.0, p< 0.001**			
(Baseline vs 90th Day)	Group II (Ortho): Paired t-test value = 22.4, p=0.002*			
Patient Hygiene Performance Index	At Baseline	2.48± 0.34	2.43 ± 0.39	0.611
	7th day	2.23 ± 0.3	2.25 ± 0.37	0.854
	21st day	1.74 ± 0.32	2.08 ± 0.28	<0.001**
	45th day	1.47 ± 0.29	1.86 ± 0.27	<0.001**
	90th day	1.11 ± 0.24	1.7 ± 0.31	<0.001**
Intragroup comparison	Group I (Ionic): Paired t-test value = 13.4, p< 0.001**			
(Baseline vs 90th Day)	Group II (Ortho): Paired t-test value = 9.87, p=0.002*			
Plaque Control Record	At baseline	51.36 ± 5.09	53.57 ± 5.75	0.157
	7th day	45.68 ± 5.90	47.16± 6.87	0.418
	21st day	34.57± 8.02	41.07 ± 8.96	0.009*
	45th day	25.48 ± 7.04	37.02 ± 9.61	<0.001**
	90th day	17.72 ± 4.38	32.04± 11.35	<0.001**
Intragroup comparison	Group I (Ionic): Paired t-test value = 29.46, p< 0.001**			
(Baseline vs 90th Day)	Group II (Ortho): Paired t-test value = 13.1, p=0.029*			
ap<0.05				

**Table 2:** Mean Microbiological analysis at baseline and 21st day -Intergroup & Intragroup comparison

***S.mutans***	**Baseline Mean ± SD**	**21st Day Mean ± SD**	**Kruskal Wallis H test**	**P value**
Group I (Ionic)	28.88 ± 29.78	3.84 ± 10.27	H = 215.0	P =0.136(NS)
Group 2 (Ortho)	70.48 ± 90.86	38.72 ±59.87	H =286.0	P =0.571(NS)
Mann Whitney U test,	U = 285 .0,	U = 150.0,		
p-value	p =0.587(NS)	p =0.001a		
*Actinomyces*	Baseline Mean ± SD	21st Day Mean ± SD	Kruskal Wallis H test	P value
Group I (Ionic)	69.36 ± 84.01	2.48 ± 7.1	H = 125.0	P =0.026a
Group 2 (Ortho)	51.76 ± 68.4	31.36 ± 49.86	H =200.0	P =0.107(NS)
Mann Whitney U test,	U =224.5,	U = 122.5,		
p-value	p =0.045a	P< 0.001aa		
Veillonella	Baseline Mean ± SD	21st Day Mean ± SD	Kruskal Wallis H test	P value
Group I (Ionic)	7.96 ± 26.9	0 (0)	H = 280.0	P =0.005a
Group 2 (Ortho)	15.32 ± 30.3	6.6 ± 29.94	H =286.0	P =0.082(NS)
Mann Whitney U test,	U =308.5,	U =262.5,		
p-value	p =0.927	p =0.039a		
p>0.05 - no significant difference, *p<0.05 - significant, **p<0.001 - highly significant
